# Bone health education in individuals with spinal cord injury or disease—the Bare Bones Podcast Series: plan it, produce it, post it!

**DOI:** 10.3389/fresc.2024.1340881

**Published:** 2024-07-16

**Authors:** B. Catharine Craven, Anita Kaiser, Lindsie A. Blencowe, Hope Jervis-Rademeyer, Lynn Boag, Wendy Murphy, Masae Miyatani

**Affiliations:** ^1^ KITE Research Institute, University Health Network, Toronto, ON, Canada; ^2^ Rehabilitation Sciences Institute, Temerty Faculty of Medicine, University of Toronto, Toronto, ON, Canada; ^3^ Division of Physical Medicine and Rehabilitation, Temerty Faculty of Medicine, University of Toronto, Toronto, ON, Canada; ^4^ Brain and Spinal Cord Rehabilitation Program, Toronto Rehabilitation Institute, University Health Network, Toronto, ON, Canada; ^5^ Canadian Spinal Research Organization, Richmond Hill, ON, Canada; ^6^ Institute of Medical Science, University of Toronto, Toronto, ON, Canada; ^7^ Department of Medicine, University of Alberta, Edmonton, AB, Canada

**Keywords:** Co-design, patients with lived experience, bone health, patient education, fracture, osteoporosis, Spinal Cord Injury

## Abstract

**Introduction:**

The Consortium for Spinal Cord Medicine's inaugural Clinical Practice Guideline for Bone Health and Osteoporosis Management for Individuals with Spinal Cord Injury or Disease (CSCM-CPG) was published in 2022 for a clinician audience. The aim of this project was to develop a podcast series to ensure people with lived experience with Spinal Cord Injury or Disease (PLEX) understand the CSCM-CPG content and know how to act to reduce their fracture risk.

**Methods:**

The “Bare Bones Podcast Series” consists of nine episodes; one related to each CSCM-CPG chapter. The podcast content and the questions asked in each podcast were co-developed by PLEX partners (PLEX-P) and the project team. Two PLEX-P acted as co-hosts for the series. The invited speaker(s) were CSCM-CPG expert panel members who participated in an informal dialogue with the hosts. Each podcast closes with a specific action a listener can do to advance their bone health. The related Educational Action Planning Tool (EAT) handouts contain text and infographic information specific to each podcast episode and include key concepts and a specific actionable take-home message. Local PLEX reviewers (PLEX-R) were invited to review podcast episodes and EATs and provide their feedback through focus group participation or one-on-one (1:1) interviews. The project team revised the podcast episodes and the EATs based on feedback from the PLEX-R prior to releasing them online.

**Results:**

Nine podcast episodes and related EATs were designed and created collaboratively with 3 PLEX-P, 22 PLEX-R, 11 CSCM-CPG expert panel members, and the project team. The episodes were titled: “Introduction to the Bare Bones of Bone Health”; “Fracture 101”; “Blood Tests—a Window into You”; “I See Your Skeleton”; “Vitamin D for all, Calcium for Some”; “Get Moving and Loading”; “Pills or Poisons & Atomic Habits”; “Snap and Crack”; and “Directions for Research”. The Bare Bones Podcast Series was shared through the project website.

**Conclusions:**

The podcasts will aid PLEX and their family caregivers to advocate for ongoing bone health assessments and to promote an ongoing dialogue with care team members regarding how to prevent fractures and fracture-related morbidity and mortality.

## Introduction

There is a compelling need for individuals with Spinal Cord Injury or Disease (SCI/D) to understand the etiology of fragility fractures and to actively work to mitigate the adverse health consequences of fractures. Fragility fractures are defined in spinal cord injury (SCI) as those that occur after a fall from standing or seated height, or less, or in the absence of trauma such as during routine activities of daily living (1). Fragility fractures are common problems that increase the morbidity and mortality of individuals with SCI/D (PLEX). In the first 12–18 months after SCI, individuals with motor complete injury experience substantial (30%–50%) declines in bone mass of the hip and knee regions (distal femur and proximal tibia regions), predisposing them to a lifetime of increased risk of lower extremity fracture. Fractures of the proximal tibia, distal femur, and hip regions are the most common, with the median time to first fragility fracture typically being at 8.5 years post injury among those with traumatic SCI (2). Approximately 2%–5% of individuals with traumatic SCI experience a lower extremity fracture each year, with a lifetime incidence of 25%–50% (2–5). Fracture rates vary in the SCI population between 2.14 and 3.2 fractures per 100 patient-years (4, 6–8). Women with SCI over age 50 are at higher risk of fracture than younger women, or men of any age (hazard ratio: 1.54, 95% CI: 1.12–2.11) (9). Fractures commonly occur after a fall from standing or from seated height onto a flexed knee, or from rotational stress on the distal lower extremity during routine activities of daily living (10–15).

Low bone mass and elevated fracture risk are not clinically problematic until a fragility fracture of the hip, distal femur, proximal tibia or distal tibia regions occur. Unfortunately, fractures after SCI/D do not always heal well, and many PLEX experience poor outcomes, including delayed union, non-union, limb malalignment, segmental shortening with pseudoarthrosis, or amputation. Fractures among PLEX, whether managed operatively or conservatively, result in increased secondary health complications including respiratory infections, pressure ulcers, urinary tract infections, delirium, and venous thromboembolic events (5, 6). Additional complications of fracture include: autonomic dysreflexia, pressure injuries, pin site or joint infection, spasticity (16), and shoulder pain with depression (9, 17). Carbone et al. have reported that in a study on a cohort of 12,389 male veterans with traumatic SCI for at least 2 years, lower extremity fractures were associated with increased 5-year mortality. The risk of mortality was greater in men over 50 years of age (HR: 3.42, 95% CI: 2.75–4.25), men with motor complete injury (HR: 3.13, 95% CI: 2.19–4.45), and men with a high Charlson Co-morbidity Index (HR: 1.11; 95% CI: 1.09–1.13) (4).

Thus, it is crucial that patients and their care providers take a proactive approach to promote bone health, and prevent fracture and limit fracture-related health complications and impact on life expectancy when fractures occur given fracture-related morbidity (5) and mortality (4, 18). Bone health experts have collaborated to develop and publish three consensus documents regarding bone health evaluation and management following SCI/D: (1) International Society for Clinical Densitometry (ISCD) Position (2) Statement (19); (2) the Consortium for Spinal Cord Medicine's Bone Health and Osteoporosis Management in Individuals with Spinal Cord Injury Clinical Practice Guidelines for Healthcare Provider (CSCM-CPG) (1); and (3) the Orthopaedic Trauma Associations (OTA) Delphi Consensus on Fracture Management after SCI (20). These consensus recommendations represent collaborative efforts among healthcare professionals to improve bone health, by facilitating recognition and management of osteoporosis, fracture risk, and fracture diagnosis among PLEX. We recognize the need to bridge the gap between the guidance for clinicians and the ability of PLEX to understand, promote, and adhere to the new CSCM-CPG and related consensus documents.

Education to improve a patient's health literacy is a key to improving osteoporosis screening and/or treatment rates (21). Poor health literacy negatively impacts patients’ health outcomes (22, 23) and affects key decision-making (24). Low health literacy can lead to physician communications being poorly understood, resulting in incomplete self-management and responsibility for bone health, as well as incomplete health service utilization (25). Common issues associated with current patient education materials include the following: (1) the majority of online health information lacks quality evidence, (2) the materials developed by healthcare professionals often overlook important information needed by end-users, and (3) the materials may not be easy to access and/or user-friendly.

Patients tend to seek information, motivation, and support for healthy living and management of their health conditions via websites (21, 26, 27). Online health information is easily accessible, with a vast amount of information in a variety of formats that can help people stay up to date with emerging information about their health conditions, and websites can facilitate shared decision-making between patients and their healthcare providers (28). Unfortunately, much of the current online health information is not based on high-quality evidence and is therefore not credible (29–32).

“Co-design” has been introduced widely in the field of patient education and engagement. Co-design is a process in which targeted end-users and other relevant stakeholders form a partnership with researchers and work together on all aspects of intervention development, from understanding the needs of end-users, to content development, and pilot-testing of project outcomes (33). Involving patients in the co-development of educational materials improves the quality of existing and future health services, and empowers the patient to ask questions (34). Studies show patient-focused education materials have led to improved clinical outcomes (35). A systematic review reported patient education is an effective strategy to improve osteoporosis screening and/or treatment rates in the able-bodied population (21). Despite the recent value placed on health education materials co-developed by patients, there are few co-developed education materials in rehabilitation settings outside of the Veterans Administration and some of the more recently funded Craig H. Neilsen Foundation projects (36).

Finally, paper forms of patient education handouts or pamphlets have been a preferred method of sharing and obtaining information due to their convenience and availability (37). However, criticisms of brochures include concerns regarding the use of medical jargon and the high literacy level needed to comprehend the material. Krontoft conducted a survey to investigate patient experiences and preferences for different forms of education materials among able-bodied patients (38). The study found that most respondents (86.46%) would like a text-based format to be available; however, half of the respondents (50.21%) also wished for an audio–visual format, followed by approximately one-third (31.67%) who desired an audio format. Patient preferences for education materials vary with age and education level. However, the majority of respondents preferred to use combinations of written, audio, and video material.

To address the perceived need for relevant and actionable education materials, we planned to use co-design methods to develop the “Bare Bones Podcast Series”, a collection of educational podcasts and related handouts based on scientific evidence, intended to be user-friendly, using clear and simple language (handout) and audio (podcast) formats that are accessible to PLEX and their family caregivers throughout North America. These podcasts and handouts are intended to aid PLEX to better understand their bone health, fracture risk and to provide education and context to PLEX prior to meeting with their healthcare provider. This ensures PLEX are well prepared with questions and expectations regarding bone health screening, treatments, fracture recognition, and options for fracture management. Shared decision-making is an essential component of bone health and fracture management for PLEX, and a working knowledge of the available therapies can help drive best-practice implementation.

A podcast is a combined digital audio file of speech, music, broadcast material, etc., made available on the internet for streaming or downloading to a computer or portable media player (39). It is similar to the traditional radio, except it is available on demand. Podcasting is a convenient and portable way to share knowledge as listeners can connect at their leisure. Educational podcasts are relatively inexpensive to produce and are among the most popular types. The format of a podcast is engaging and allows for active listening during leisure time or physical activity. Podcast series typically include an introductory episode, followed by content episodes, and a final episode that often includes a wrap up or series highlights. In the field of SCI/D, podcast series for several topics are available such as Activity-Based Therapy (40), American Spinal Injury Association (ASIA) SCI Science Perspectives (41), and International Spinal Cord Society (ISCoS) podcasts (42).

A handout, by definition, is an unbound leaflet used to provide health information on a single subject. Handouts are cheap to produce and can be readily distributed to PLEX in paper and e-formats suitable for distribution in hospital and community settings. We use the term Educational Action Planning Tool (EAT) throughout this article to describe the content and utility of podcast-related handouts.

Patient-centered care has its roots in the disability movement, which aims to change healthcare from within by facilitating partnerships among patients, families, and healthcare professionals and is based on the premise that informed patients are better able to advocate for appropriate and timely care (43). The Bare Bones Podcast Series comprises a podcast series and related EATs. The Bare Bones Podcast Series seeks to advance patient-centered care by empowering PLEX to be their own advocates. This includes making positive lifestyle choices to augment their bone health, selecting appropriate treatments, and reducing the prevalence of fractures and fracture-related morbidity and mortality. We hypothesized that the Bare Bones Podcast Series would be an effective means to educate PLEX and their family caregivers about bone health, osteoporosis, and fracture risk.

## Methods

### Methods overview

Our podcast and related EAT content were based upon the CSCM-CPG for healthcare providers, which is the most comprehensive clinical practice guideline (CPG) covering detection and management of low bone mass, osteoporosis, and fracture for PLEX among recently published consensus documents (1, 19, 20). The target audience for the Bare Bones Podcast Series was PLEX, their family, and caregivers living in North America who may not possess strong foundation knowledge related to bone health. The target audience for dissemination includes PLEX affiliated with the Paralyzed Veterans of America (PVA) and other SCI-specific non-governmental organizations, including ISCoS, ASIA, and the Canadian Spinal Cord Injury-Rehabilitation Association (CSCI-RA). The project team included three PLEX partners (PLEX-P) with prior media appearances and advocacy training who assured the content was relevant, and four project team members with scientific and methodological expertise in sublesional osteoporosis, diagnostic imaging, biochemistry, clinical research, podcast development, and knowledge translation related to PLEX. The project also involved the engagement of the CSCM-CPG Expert Panel members as episode guests and PLEX reviewers (PLEX-R) who were not part of the project team but contributed to reviewing the podcasts and EAT content.

In collaboration between PLEX and members of the CSCM-CPG Expert Panel, the objectives of this project were

1. to co-develop a series of nine freely available podcasts and related EATs that are accessible to PLEX, their family, and caregivers; and
2. to disseminate podcasts and EATs via an accessible website.

This project was approved by the University Health Network (UHN) Quality Improvement Review Committee. As the project falls outside the scope of research requiring Research Ethics Board (REB) review, ethics approval was waived by the committee and confirmed by a UHN REB Chair.

**Figure 1:**
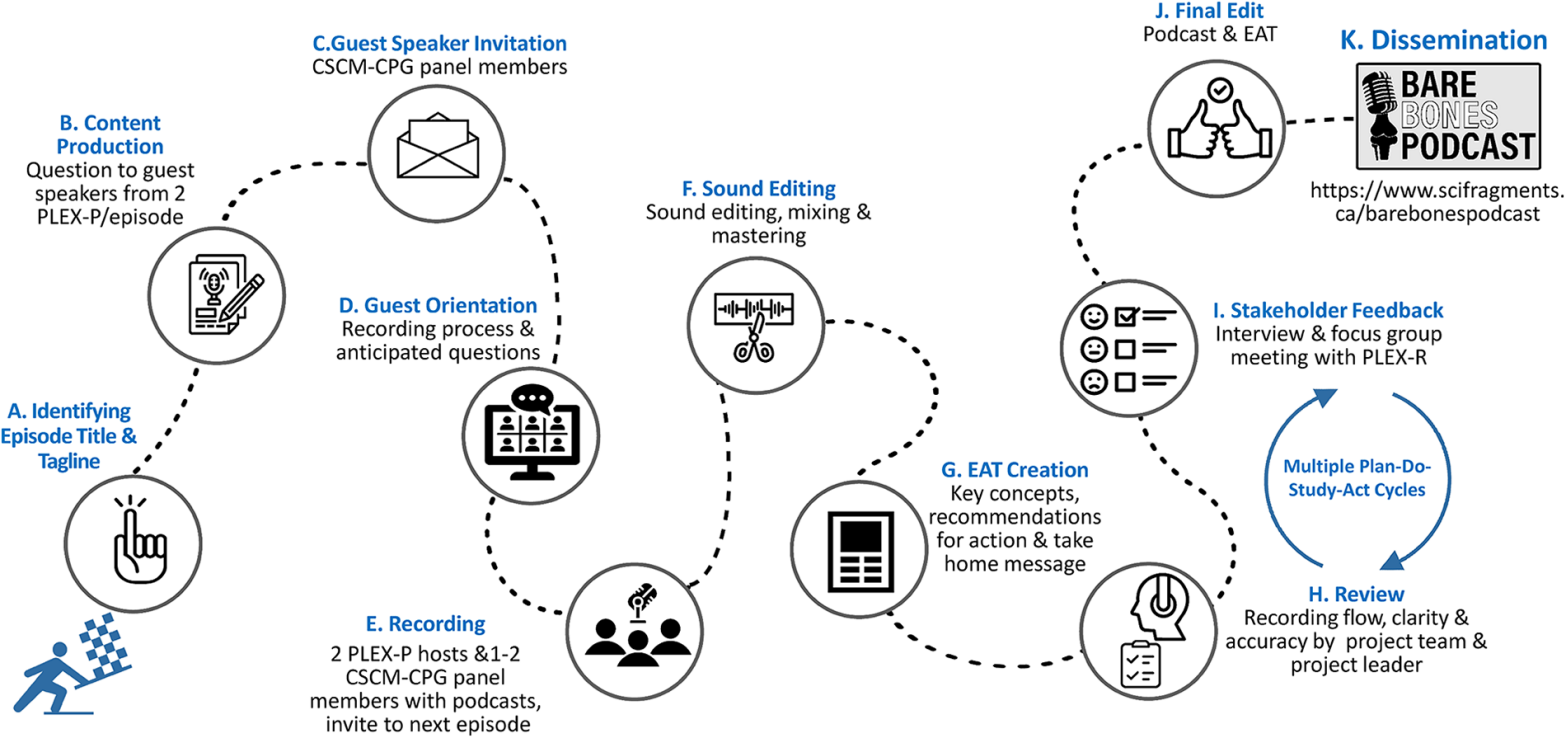
Bare bone series development and evaluation process map. (**A**) Identifying Episode Title & Tagline; (**B**) Content Productio; (**C**) Guest Speaker Invitation; (**D**) Guest Orientation; (**E**) Recording; (**F**) Sound Editing; (**G**) EAT Creation; (**H**) Review; (**I**) Stakeholder Feedback; (**J**) Final Edit; (**K**) Dissemination.

At the project outset, the team convened regularly to compile and assemble the podcast and EAT content. A statement of work for each PLEX-P was created for their role at the outset of the project. They received payment bi-annually for their contribution to the project. Figure 1 displays the Bare Bones Series Development and Evaluation Process Map. Briefly, the podcast key questions were identified and planned by the project team (Figures 1A,B). Podcast guests were oriented to the content and recording process. Each podcast was recorded featuring two PLEX-P acting as co-hosts and one or two CSCM-CPG authors/panel members as invited guests (Figures 1C–F). EATs were created to correspond with each podcast episode (Figure 1G). The project leader and team reviewed each podcast and EAT for clarity and accuracy of the content and alignment with the CSCM-CPG (Figure 1H). PLEX-R were recruited to review and provide their feedback on blocks of at least three podcast episodes and related EATs (Figure 1I). The project team revised and refined the podcast content and EATs based on the feedback from PLEX-R (Figure 1J). The finalized podcast episodes and EATs were disseminated via the project website (Figure 1K).

### Podcast episodes

#### Planning the podcast

The podcast series structure was intentionally developed to mimic the CSCM-CPG Structure (Figure 1A). Team members (BC, MM, AK, HJ-R, LB, LAB, and WM) created a table outlining the number of episodes, title of each episode, key concepts, learning objectives, and episode descriptions. The opening taglines give listeners insights into the upcoming discussion with the episode guests, while not disclosing the actual take-home message. After each episode, listeners are provided with a take-home message or helpful tip they can keep in mind or act upon to advance their bone health. The taglines and take-home messages are designed to keep listeners engaged, in not just one episode of the podcast but the full series of informative topics, and to support the flow of information throughout the series regarding achieving healthy bones and a fracture-free life.

#### Generation of the interview questions

All questions posed in the podcasts were generated by PLEX-P (LB, AK, and WM) after reviewing the nine CSCM-CPG chapters (Figure 1B). The PLEX-P chose chapters they wished to review. Each PLEX-P was responsible for three chapters each. Their task was to select/generate pertinent questions that they felt adequately reflected the CSCM-CPG content and were important to managing bone health after SCI/D. Each PLEX-P created a list of questions for the chapter to be reviewed and shared with the group as a mock up for the podcast discussion. From the generated list of questions, the most relevant questions were selected and refined for simplicity using plain language and posed in a conversational format during the podcast interviews.

#### Recruitment of expert guest speakers

Altogether 11 expert CSCM-CPG members from Canada and the US were invited to participate as guests in the Bare Bones Podcast Series (Figure 1C). All invited experts agreed to the podcast participation, with five of the nine episodes having two guest speakers. Prior to podcast recording, the guests completed a written consent form and were provided an information package outlining the episode structure along with the introductory tagline, list of potential interview questions, and take-home message to review specific to their expertise. Guests were provided a list of equipment they would need and instructions on how to join the web-based platform (Zoom, Zoom Video Communications Inc., San Jose, California, United States) and achieve clear sound during the recording. The guest speakers participated in a 1-hour planning meeting, led by the PLEX-P podcast hosts (AK and WM) and the co-producer (HJ-R) who handled the technical aspects of the podcast (Figure 1D). During the planning meeting, the co-hosts reviewed and discussed the material with the guest speakers, and revisions were made to the interview questions, introductory tagline, and take-home messages to reflect the information exchanged and focus on the most salient points. The co-producer (HJ-R) conducted an audio test and reviewed the equipment and set-up, and shared tips to produce high-quality sound.

#### Interview script development

Following the planning meeting, team member and podcast host (AK) converted the interview questions, introductory tagline, and take-home message for each episode into an interview script. Team member and co-host (WM) and the project leader (BC) reviewed the episode scripts and refined as needed. The script dialogue was framed in a conversational tone and worded in plain language. Scripts for each podcast episode followed a similar format: introductory tagline, introduction of co-hosts and guest(s), interview questions guided by hosts, take-home message, and information about the next episode, and where to locate the episode-related EAT.

#### Podcast recording

Prior to recording sessions, guests were sent email reminders regarding the equipment needed, instructions on how to log in to the Zoom and an audio checklist (Figure 1E). The guests were provided the episode script and asked to prepare talking points for the interview questions, keep the responses succinct, and providing responses in plain language while allowing for conversational dialog.

At the time of recording, the project team (AK, WM, and HJ-R) met with guests to orient them again to the recording process, do a dry run of the interview, and answer any questions they had prior to recording. The entire recording process lasted approximately 1-hour. The series hosts (AK and WM) alternated between the primary and secondary co-host roles across the podcast series. Episode one was recorded first as a pilot episode with project leader and guest (BC). The remaining episodes were recorded according to guest schedules and availability.

#### Sound editing and production

To develop the podcast theme music, the co-producer (HJ-R) presented four sound samples for the team to consider (Figure 1F). The project team selected three themes that were mixed into a sample introduction. After listening to the sample introductions, the team chose the podcast theme music.

Once recording of an episode was completed, sound edits were made using GarageBand 10.4.8 (Apple Inc., Cupertino, California, United States) while referencing the transcripts. After sound editing, the episode recording was sent to the podcast team (AK, BC, MM, WM, LB, and LAB) to review and provide content and technical feedback. The co-producer (HJ-R) made edits based on the feedback and the podcast was then reviewed by the executive producer (BC) before the episodes were evaluated during focus group discussions and one-on-one (1:1) interviews with PLEX-R.

#### Video editing and production

Once the sound engineering was completed for each episode, video production began. The co-producer (HJ-R) used iMovie 10.3.5 (Apple Inc., Cupertino, California, United States) to create episode videos. A slideshow was developed for each episode to align with the podcast audio. In general, the slide shows contain the episode number, logo for the Bare Bones Podcast Series, host and presenter biographies, key information (e.g., episode series content), funding, and credits. The videos were reviewed by the podcast team prior to release.

#### EAT handouts

An EAT was developed for each podcast episode using lay language targeting a Grade 8 reading level. Each EAT contains the corresponding podcast title, key concepts, background information, and recommendations for action, podcast link, and additional resources (Figure 1G). The information in the EAT was presented using a mix of text and infographic information. Multiple Plan–Do–Study–Act (PDSA) cycles (44) were completed to iteratively refine the EATs. This varied from 10–30 versions per EAT.

#### Podcast episodes and EATs internal review

The project leader and project team reviewed each podcast and EAT for content, clarity, flow, accuracy of the content, and CSCM-CPG content alignment prior to their evaluation by the PLEX-R (Figure 1H).

#### Bare bones podcast series evaluation

PLEX-R were recruited from a tertiary SCI rehabilitation center in Toronto Canada (Lyndhurst Centre, UHN) (Figure 1I). We intended to recruit 8–10 participants per evaluation block. The consenting PLEX-R reviewed a block of three episodes and related EATs. The participants spent 1 hour reviewing the three podcasts and the three related EATs prior to participating in either a focus group or 1:1 online interview. The focus group and interviews were conducted with three different groups of PLEX-R on three separate occasions with iterative edits throughout. During the focus group meeting, the EATs were shared on screen with the PLEX-R through Zoom's screen share function. The focus groups were conducted by the project leader, project coordinators, a PLEX-P (LB), and two to five consenting reviewers. The focus groups lasted 60–90 min and took place through Zoom (Zoom Video Communications Inc., 2016). Each meeting was opened with introductions, a short project overview agenda, and quick overview of Zoom functionality. The focus group meetings were conducted using a semi-structured interview guide that included qualitative open-ended questions and closed questions to identify trends in terms of (1) design, structure, and format; (2) terminology and word choices; (3) presentation of key concept; (4) action items and take-home message; (5) cultural-linguistic acceptability; and (6) knowledge translation as well as to assess PLEX-R knowledge pertaining to the material discussed (see Supplementary Material: One-to-One Interview and Focus Group Meeting Guide).

The 1:1 interviews were conducted by a PLEX-P (LB) using Zoom. The interviewer received training by conducting a mock interview with an experienced research coordinator who was not involved in the project. Interviews were 30–60 min in length and used the same questions as were posed in the focus group meetings.

All PLEX-R received a gift card and thank you note for their contributions to the project after their the focus group or 1:1 interview participation.

All focus group meetings and interviews were recorded and recommendations were summarized for the project team to review. The project team thoroughly examined and discussed the recommendations from the PLEX-R, aiming to delineate common patterns and identify novel ideas within each group and to summarize overarching observations.

Feedback collected from PLEX-R was used to identify unclear content in the podcasts and EATs and to clarify preferences for the visual formatting of the EATs. This informed substantial revisions to the EATs, as well as to the podcasts and dissemination plans.

#### Final edit

The podcast episodes were finalized and the EATs were converted to pdf files to upload on to the project website (Figure 1J).

#### Website and podcast episodes and EATs dissemination

Website development began as a team brainstorming session and a subsequent website framework was created. The website was envisioned as a repository of SCI bone health information, which would include the podcast and other resources. The project team then reviewed other health-related podcasts and associated websites, to further refine the website framework and develop a preferred design based on esthetic feedback and functionality. Website building platforms were investigated. WIX is regularly listed as one of the top three website building platforms and it had the functionality to accommodate the features outlined in our framework for a reasonable fee, specifically YouTube videos, audio streaming, PDF downloads, website analytics to track use, and Google search engine optimization (SEO), which would enable the podcast to appear in Google search results. WIX online tutorials and video training resources helped guide website design and production (45, 46). The domain name www.scifragments.ca was selected, purchased, and connected to the WIX site (Figure 1K).

Once the podcast episodes were prepared and ready to release (audio and EATs finalized and accompanying slide show created), a YouTube podcast was created. Online YouTube tutorials guided this process (46). As a YouTube podcast, the audio content is available on YouTube Music, while the videos are also available on a YouTube channel. The videos, download links for the EATs, and links to YouTube Music were then added to the website.

Survey Monkey was used to create Feedback Questionnaires. Survey Monkey provides guidance on survey creation (47). The survey links were added to the website to collect users’ feedback and to assess users’ knowledge pertaining to the material discussed in the podcast and EAT. The survey questions were designed to ascertain the following information:

(1) Are end-users absorbing key information? (Can they identify and report back specific key learning objectives?)
(2) How likely are PLEX to incorporate material from the EAT/podcast into daily life or use it to self-advocate with their healthcare team? If yes, how often?
(3) How likely are PLEX to share the EAT/podcast or its learnings with others (family, caregivers, healthcare providers)?

The final component of our website development was the addition of Google Analytics to track use of the website. Google's Analytics Academy training program provided the necessary training to understand and implement Google Analytics on the website (48). While WIX does have analytics features built into the platform, Google Analytics is more comprehensive.

## Results

### Bare Bones Podcast Series

As mentioned previously, nine podcast episodes and related EATs were designed and created collaboratively with the 3 PLEX-P, 22 PLEX-R, and 11 members of the CSCM-CPG expert panel and the project team (Table 1). Based on the feedback from PLEX-R, Episode 8 was divided into Part 1: Warning Signs of Fracture & Fracture Management and Part 2: Rehabilitation & Osteoporosis Therapy after a Fracture. Each podcast episode was 8–15 minutes in length. Related EATs were one page per episode for Episodes 1–4, 6, 7, and 9. Episode 5 (Calcium and Vitamin D) was two pages based on PLEX-R feedback (See next PLEX-R feedback section). Episode 8's EAT was created for Part 1 and Part 2 separately (Table 2).

**Table 1:** Summary of project team and collaborators.

Group	Role (initial)
Project team (*n* = 7)	Executive producer (BC)
	Podcast co-producer (HJ-R)
	Podcast co-hosts (AK and WM)^a^
	Content project team (LB, WM, AK, and MM)^a^
	Evaluation team (LB and MM)^a^
	EAT creators (MM, BC, and LAB)
	Website producer (LAB)
CSCM-CPG expert panel (*n* = 11)	Podcast guest speakers
PLEX-R^b^ (*n* = 22)	Podcast episode and EATs reviewers

^a^ AK, WM, and LB are PLEX-P.

^b^ PLEX-R were PLEX reviewers who were recruited from the local outpatient clinic.

**Table 2:** Summary of Bare Bones Podcast episodes.

Episode No.	Episode title	Take-home messages	Episode length	CSCM-CPG chapter
1	Introduction to the Bare Bones of Bone Health	Knowledge is power. See the complete picture for healthy bones and a healthy life.	10:49	Not applicable
2	Fractures: 101	Know your fracture risk. Act to change modifiable risk factors.	11:32	1. Medical History, Assessment of Fracture and Fall Risk
3	Blood Tests: A Window Into You	Get a blood test to learn about your bone health.	7:26	2. Laboratory Screening
4	I See Your Skeleton	Get your knee region bone density test on a regular basis to monitor your bone health and your fracture risk.	13:40	3. Bone Density Testing with Dual-Energy X-ray Absorptiometry 4. Volumetric Bone Density and Bone Architecture: Peripheral Quantitative Computed Tomography and Quantitative Computed Tomography
5	Vitamin D for All, Calcium for Some: Bones, Groans, and Stones	Follow our doctor's advice to get sufficient amount of dietary calcium and vitamin D supplement.	12:37	5. Calcium and Vitamin D3: Diet or Supplements
6	Get Moving and Loading	Exercise is good for you, but passive standing and electrical stimulation-based activities can increase bone health in your legs.	14:48	6. Rehabilitation Therapy
7	Pills or Poisons, and Atomic Habits	There are drugs available to prevent and treat bone mineral density decline. Talk to your doctor about the best drug for you.	14:58	7. Drug Therapy
8 part 1	Snap and Crack Part 1: Warning Sign of Fracture & Fracture Management	Know the warning signs of fracture and seek urgent care from an orthopedic surgeon.	15:32	8. Fracture Management
8 part 2	Snap and Crack Part 2: Rehabilitation & Osteoporosis Therapy after a Fracture	If you have a fracture, get advice from your rehab team to restore your functional independence and to prevent future fracture.	9:55	
9	Directions for Future Research	Consider partnering with researchers to reduce fractures.	11:46	9. Directions for Future Research

### PLEX-R feedback and podcast and related EAT revision

Altogether 31 PLEX with chronic SCI/D living in Ontario consented to review the podcasts and EATs and participate in either a focus group meeting or a 1:1 interview. Nine of the 31 withdrew their consent due to medical reasons or time pressures. A total of 22 PLEX-R were engaged in the review process of whom 17 were men and 5 were women. The PLEX-R included individuals with paraplegia, tetraplegia, and high tetraplegia and those who walk or use a wheelchair for household and community mobility. The number of unique focus group participants or 1:1 interview participants is provided in Table 3 as the episodes and EATs were bundled for review. The feedback on the podcast episodes and related EATs and the associated revisions are summarized in Table 4. The Supplementary Material (EAT Diagram) shows the EAT diagram created based on PLEX-R feedback.

**Table 3:** Summary of PLEX-R participants of focus group meetings and one-to-one meetings.

Group	Reviewed podcast	PLEX evaluators (men = 17, women = 5, Total *n* = 22)
	Episode and EATs	
1	1, 2, and 4	Focus group, *n* = 4
		1:1 interview, *n* = 4
2	3, 5, and 6	Focus group, *n* = 4
		1:1 interview, *n* = 5
3	7, 8, and 9	Focus group, *n* = 2
		1:1 interview, *n* = 3

**Table 4:** Summary of feedback from PLEX-R on the podcast episodes and related EATs and revisions made based on the feedback.

Category	Examples of issues and suggestions	Action taken
Design, structure, and format	Need to orient PLEX to the EAT format and use	Diagram outlining EAT Content was added to the Website (see Supplementary Material: EAT diagram)
	Highlight related episodes within the podcast and EAT to assist users to find more information	Related episodes referred to one another within podcasts and EATs (i.e., calcium and vitamin D intake Episode 5 referred to the lab testing episode 3)
	Requests to visually simplify the EAT in terms of number of infographics and use of color	The color background was simplified in EAT 1–9. Numbers were added to key concepts in EAT 3–9 to help the reader navigate
	Request to re-record podcast interview with a more natural conversation	Podcast episodes 1 and 8 were re-recorded
	Reduce volume of information in podcast and EAT	The EAT 5 was expanded into two pages to reduce volume of information on each page. EAT 4 was simplified with a reduction of a number of infographics used. In EAT 9, the number of key concepts was reduced to two from four
Terminology and word choices	Limit the use of medical terminology and use lay language	The language was simplified in each EAT. Some terms were intentionally not simplified as PLEX-R highlighted to need to use the same language with their healthcare providers (e.g., EAT 3, blood test items)
Presentation of key concept, key action items, and take-home message	Ensure the EAT action items and take-home messages are clear and written in an authoritative voice	The take-home messages were visually emphasized in EAT 1–9. Take-home messages for episodes 5, 8, and 9 were refined
	Emphasize the importance of consulting with healthcare providers before implementing recommendations	The need to consult a regulated healthcare profession were emphasized in EAT 2–9
	Information should be updated when the CSCM-CPG is updated	OFI for future revisions
Cultural-linguistic acceptability	Podcast and EATs are acceptable culturally and linguistically	Translate EATs to other languages as OFI
Knowledge translation	Importance of information shared in the series was identified	OFI during dissemination
	Synergistic benefits of the combination of podcast and EAT were reported	
	Create and distribute magnets or bookmarks to promote the Bare Bones Podcast Series	Magnets and bookmarks were created and are being distributed (e.g., at scientific conferences)
	Present a slide show about the Bare Bones Podcast Series in clinic waiting areas	OFI during dissemination
	Format print EATs as single pages or booklet depending on users’ preferences	We are experimenting with distributing EAT booklets or leaflets

OFI, opportunity for improvement.

### Podcast and EAT dissemination

We disseminated the finalized podcast and related EATs online from the project website (https://www.scifragments.ca/barebonespodcast) and announced the dissemination in person at the Academy of Spinal Cord Injury Professionals (ASCIP) meeting on 5 September 2023. The Bare Bones Podcast Series was introduced during the presentation. Three-hundred magnets including the project website and common sources of dietary calcium were distributed at the North American Spinal Cord Injury Consortium (NASCIC) booth at the conference. The project X account (@SCIBare Bones) was created to provide a venue to share and discuss our podcast episodes and EATs and bone health–related information and events with patients and family caregivers and several networks including PVA, Spinal Cord Injury Research Evidence (SCIRE), PRAXIS, and the Ontario SCI Alliance and the informal networks of each CPG panel member. We have posted a survey for ongoing feedback regarding the EATs and podcast episodes.

## Discussion

This project aimed to co-develop and disseminate a series of nine educational podcasts and related EATs, in a series titled “The Bare Bones Podcast Series”. Nine podcast episodes and related EATs were designed and created collaboratively with the project team including three PLEX partners (PLEX-P), local PLEX reviewers (PLEX-R), and members of the CSCM-CPG Expert Panel. The Bare Bones Podcast Series was disseminated through the project website and promoted at scientific conferences and through a social media campaign.

In the present project, PLEX-P contributed, collaborated, or were empowered to do the following: (1) participate in grant development and the funding application; (2) identify and refine the podcast titles, opening taglines, key concepts, and take-home messages; (3) schedule and organize planning meetings for guest speakers; (4) host podcast episodes and direct the discussion for each podcast episode; (5) contribute to the creation interview guides; (6) conduct focus groups and 1:1 interviews with PLEX as interviewers; (7) review each podcast episode and EAT for flow and clarity; (8) provide advice regarding the content and language used and ensure consistency between the content of CSCM-CPG and that of the podcast and EATs; (9) provide feedback on the website structure and content and contribute to the dissemination of ideas; (10) contribute as co-authors for conference presentation and workshops; (11) serve as subject-matter content experts and provide advice regarding the education materials’ relevance to others with lived experience.

A total of 22 PLEX-R acted as podcast and EAT reviewers. Project team members collected information regarding how education materials should be structured and delivered. This enabled the project team to discern reviewers’ perceptions of the podcast episodes and EATs, their efficacy, and ways to improve our dissemination strategy in an impactful way. Although CPGs are traditionally developed for healthcare providers, they can provide useful information to PLEX, family caregivers, informal and formal caregivers, as well as other members of the public (49).

### Bone health education for PLEX

Crack et al. conducted a cross-sectional survey among 138 adults with SCI/D, regarding their knowledge and awareness of post-SCI bone health. Self-reported assessments of bone health knowledge were analyzed. Among Canadian participants, 30% (*n* = 42) believed they had adequate knowledge on bone health, while 70% (*n* = 96) believed their knowledge was inadequate or were unsure. Most participants (73%, *n* = 101) reported being concerned about the risks of low bone mineral density (BMD) after SCI and were interested in learning more about prevention (76%, *n* = 105) and treatment options (78%, *n* = 108) (50). Further, Etingen et al. interviewed 32 US Veterans with SCI/D who had experienced at least one lower extremity fracture in the prior 18 months to describe the patients’ pre-fracture knowledge of osteoporosis and bone health, diagnosis and management of osteoporosis, history and experiences with fracture treatment, and post-fracture care and experiences (18). The results suggest individuals with SCI/D may lack knowledge about bone health and fracture prevention, and following fracture, feel unable to resume pre-fracture participation. In addition, individuals with SCI/D reported they did not feel engaged when establishing fracture treatment plans. These results imply that individuals with SCI/D could benefit from education regarding bone health and fracture prevention and management. Topics of interest identified included screening, lifestyle modifications, drug/rehab therapy for low bone mass/osteoporosis prevention and treatment, fracture treatment options, and considerations of subsequent function and participation. The Bare Bones Podcast Series attempts to address some of the articulated needs.

Further, Nayak et al. conducted a systematic review and meta-analysis of the efficacy of quality improvement strategies to improve osteoporosis screening [BMD/dual-energy x-ray absorptiometry (DXA) testing and/or treatment (pharmacotherapy)] initiation rates in the general population (21). The results showed patient education/activation appear to be effective for improving BMD/DXA testing and/or osteoporosis treatment rates in patient populations with recent or prior fracture. For populations that include individuals without prior fracture, the results indicated that patient self-scheduling of DXA appears to be an efficacious strategy to increase DXA testing rates. The results of this systematic review among the able-bodied population reinforces that patient education is likely a beneficial strategy to improve osteoporosis screening and/or treatment rates for the SCI/D population.

### Podcast and EAT development

Our patient education materials have three main strengths: First, we created evidence-based patient education materials. The podcast and related EAT content were based upon the CSCM-CPG for healthcare providers, which was developed based on evidence that was systematically and scientifically obtained. Second, the podcast and related EAT were co-designed and developed collaboratively with researchers, PLEX, members of the CSCM-CPG panel, and the project team to ensure the materials were adapted to the specific needs and perceptions of the PLEX while ensuring rigorous content. Further, pairing of the audio podcasts and provision of the visual EAT uses two different forms (i.e., audio and visual formats) so that PLEX can choose to use one or both formats to aid their learning and reinforce their recall of the material. These EATs are freely available through the project website, and may be shared freely; however, permission is required to alter them.

1. Evidence-based patient education materials

Health information that lacks quality evidence is unlikely to produce the desired health benefits and may have adverse effects on health outcomes (51). Unfortunately, the general public lacks the skills necessary to distinguish evidence-based resources from those that are not trustworthy (52). The EATs were derived from the information within the CSCM-CPG, which was derived from a series of systematic reviews or narrative reviews based on a comprehensive search of the latest evidence and collaborative synthesis of the related data within the CSCM-CPG panel, the International Society of Clinical Densitometry position statement lead, and the Orthopaedic Trauma Association Delphi consensus lead to align recommendations where feasible. To help users understand that the Bare Bones Podcast Series is credible, the first page of the project website has a brief statement asserting that the Bare Bones Podcast Series is based on the content of the CSCM-CPG and provides the CSCM-CPG link for sharing with one's healthcare provider. Users can visit the expert page listing within the website so they know who contributed to the project and approach local experts where feasible. Episode 1 of the podcast outlines the CPG definition, purpose, and development processes in a simple language to help the listener understand that the Bare Bones Podcast Series content was developed based on the CSCM-CPG recommendations.

2. Co-designed patient education materials

Patient educational materials are often developed by healthcare providers and hence potentially miss important information needed for end-users. To address this issue, we co-designed the Bare Bones Podcast Series and developed patient education materials (i.e., podcasts and EATs) with PLEX to ensure their relevance and effectiveness. Among the multiple strengths of our podcast development process described earlier, a noteworthy strength is that the questions asked in each podcast were co-developed by PLEX partners (PLEX-P), and the podcast hosts were also PLEX-P who share similar experiences and life situations with the intended listeners/users. Additionally, during the development of the podcast and EATs, we received feedback from PLEX reviewers (PLEX-R) on the relevant themes and made revisions based on the feedback (Table 4). It was not possible to make every change suggested by PLEX-R as their comments and preferences differed. Therefore, we made revisions to increase knowledge, likelihood of listening/reading, and reduce barriers to learning about bone health and fracture management considering common themes or patterns to the PLEX-R feedback. For example, some reviewers suggested simplifying the name of the recommended blood test items in both podcast episodes and EATs (Episode 3: Blood tests—a window into you); while others advocated the use of medical terminology to enable the patient and provider to use the same language when discussing the EATs. Throughout our development process we focused on empowering PLEX to advocate for themselves and encouraged dialogue with their healthcare providers. Thus, we decided to keep the medical terminology regarding necessary blood tests in the Episode 3 but highlighted that patients need to discuss with their healthcare provider to ensure they have all the recommended blood tests.

3. Audio and visual formats of patient education materials

The EAT content was presented using a mix of text infographic and photographs. In the past, paper brochures or pamphlets of patient education materials have been a preferred method for distributing and obtaining information due to their convenience and availability (37). However, criticisms of brochures or pamphlets have included the use of medical jargon and high literacy level. To improve the usability and impact of text-based information, visual material was included with the text to increase understanding, an approach that is helpful for people with low literacy skills or for whom English is a second language (53–55). The use of infographics is a well-substantiated choice for EAT content. In the field of bone health education, adults aged 50 years and older who evaluated educational brochures preferred the brochures with photographs of people of varying ages (50 years and older), races, and genders, as well as photographs supporting each topic (e.g., calcium-rich foods) rather than the brochures that used simple line illustrations (56). In addition, others have reported that infographics and photographs activate visual and verbal language centers in the brain and achieve more optimal learning (57).

Podcasts are increasing in popularity in the patient education field (58–61). Users can listen to the podcast from our website without downloading them to their own device. Following discharge from the inpatient rehabilitation setting, many adults with SCI/D seek to participate in learning in a variety of settings, and many have to balance the competing pulls of self-care, work, and family time. Podcasts can help to facilitate self-paced learning for PLEX in their down time. Health-related podcasts have been shown to be effective in increasing knowledge and promoting positive health behaviors across a range of topics, including prostate cancer (60), nutrition (58), weight loss (61), and menopause (59). A recent study with able-bodied women aged 40–60 years indicated that story-based podcasts introduced by diverse audiences are an engaging and effective way to learn about menopause and change health behavior. However, positioning health information in the form of narratives may influence patient choice and distract from scientifically validated medical information (62, 63). Therefore, ensuring evidence-based content is also important when developing patient educational podcasts. All our PLEX-R indicated that the two formats (podcast and EATs combined) were helpful to understand key concepts and action items.

This study has some limitations. First, the number of PLEX-R was small and they were all from one region, which may limit generalizability. Second, PLEX-R had chronic SCI/D for periods varying from many months to decades; therefore, their feedback may not specifically reflect the thoughts and feelings of newly injured patients. In fact, a few PLEX-R commented that they could now understand the content easily and recognized the value of the information as they had osteoporosis and had been exposed to therapy. They were unsure if new patients who had not experienced screening, diagnosis, or therapy would understand the information provided and/or use the information effectively. Lastly, there were more comments on EATs than podcast episodes during the focus group meetings. Displaying the EATs on the screen may have distracted/biased reviewers.

Our podcast episodes and related EATs are intended to be living education materials that will be revised periodically. The next phases of the project will be to evaluate listeners’ perceptions of the podcast episodes and EATs and their efficacy through the online survey linked with podcast episodes and related EATs on the website. In addition, we have recorded sound bites (including Soundbites for YouTube, X, TikTok) to help spread the podcast content. The website will be promoted via social media accounts (X, Instagram, etc.) of SCI-specific non-governmental organizations (i.e., PVA, NASCIC, SCIRE, SCI Canada, SCI Ontario, SCI-Implementation and Evaluation Quality Care Consortium Praxis, etc.). The podcast series and EATs will also be promoted at PVA and UHN clinical sites and via poster/workshops at the future ISCoS, ASIA, and Canadian Spinal Cord Injury-Rehabilitation Association conferences and during Osteoporosis month campaigns.

## Conclusion

The Bare Bones Podcast Series includes nine podcast episodes, and the related EATs were co-designed and co-developed and released through the project website for PLEX to introduce them to the content of the newly published Consortium for Spinal Cord Medicine's Bone Health and Osteoporosis Management CPG. These podcasts are freely available online at https://www.scifragments.ca/barebonespodcast. We anticipate the Bare Bones Podcast Series will aid PLEX, their family caregivers, and healthcare providers to advocate for ongoing bone health screening/assessments and to promote an ongoing dialog with care team members regarding how to prevent fractures and fracture-related morbidity and mortality.

## Data Availability

The original contributions presented in the study are included in the article/**Supplementary Material**, further inquiries can be directed to the corresponding author.
