# Supplementary material for: Bone health education in individuals with spinal cord injury or disease—the Bare Bones Podcast Series: plan it, produce it, post it!
Source: Front Rehabil Sci. 2024 Jul 16;5:1340881. doi: 10.3389/fresc.2024.1340881 (PMC11286568; doi:10.3389/fresc.2024.1340881)
Supplement: Supplementary file 1 [file Datasheet1.pdf]

Supplementary Material

Bone Health Education in Individuals with Spinal Cord injury or Disease: The Bare Bones Podcast Series: Plan it, Produce it, Post it!

B. Catharine Craven*, Anita Kaiser, Lindsie A. Blencowe, Hope Jervis-Rademeyer, Lynn Boag, Wendy Murphy1, and Masae Miyatani1

*** Correspondence:**

Dr. B. Catharine Craven
cathy.craven@uhn.ca

1 Supplementary Material

- Bare Bones Series One to One Interview and Focus Group Meeting Guide
- EAT (Educational Action Planning Tool Handout) Diagram

Bare Bones Series One to One Interview and Focus Group Meeting Guide

Q1. Is the information clear and easy to understand?

- Yes
- No
- If, no, which parts are not clear and/or easy?

Q2. Is there anything offensive?

- Yes
- No
- If, yes, which parts are offensive?

Q3. What is helpful?

Helpful information includes:

Q4. What is not helpful?

Not helpful information includes:

Q5. After reading the handout and listening to the podcast, do you know what actions to take?

- Yes
- No
- If yes, what do you plan to do

Q6. How would you rate the quality of the podcast & handout as a joint product?

- Very good
- Good
- Acceptable
- Poor
- Very poor

Q7. How would you rate the usefulness of the podcast & handout as a joint product?

- Very good
- Good
- Acceptable
- Poor
- Very poor

Q8. The podcast and pamphlet presentation is a good way to make Clinical Practice Guidelines, Bone Health and Osteoporosis Management in Individuals with SCI available to the public.

- Strongly agree
- Agree
- Neither agree or disagree
- Disagree
- Strongly disagree

Q9. Any other comments or suggestions to make our products (podcast and handout) better?

EAT (Educational Action Planning Tool Handout) Diagram

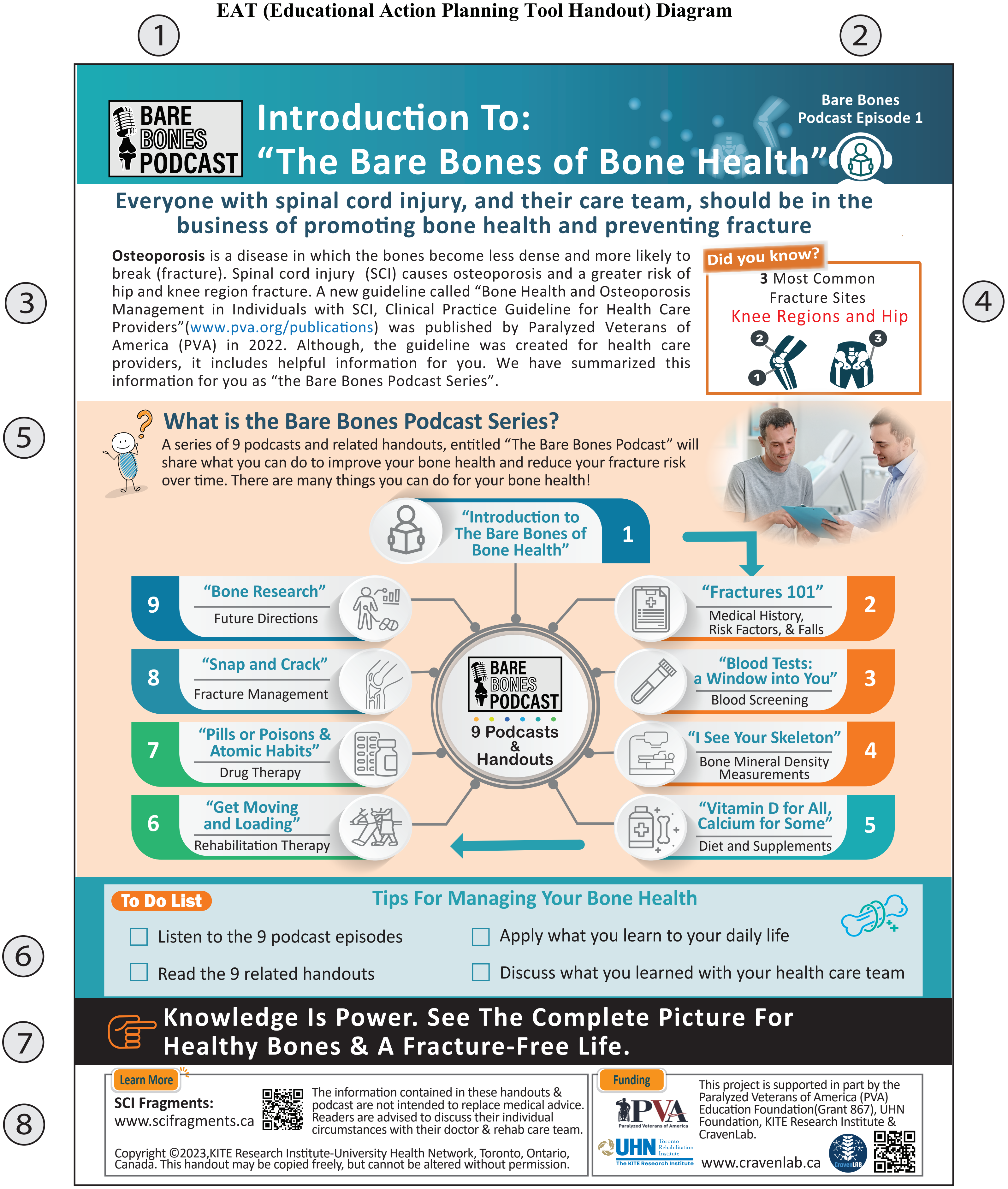

BARE BONES PODCAST

Introduction To: "The Bare Bones of Bone Health"

Bare Bones Podcast Episode 1

Everyone with spinal cord injury, and their care team, should be in the business of promoting bone health and preventing fracture

Osteoporosis is a disease in which the bones become less dense and more likely to break (fracture). Spinal cord injury (SCI) causes osteoporosis and a greater risk of hip and knee region fracture. A new guideline called "Bone Health and Osteoporosis Management in Individuals with SCI, Clinical Practice Guideline for Health Care Providers"(www.pva.org/publications) was published by Paralyzed Veterans of America (PVA) in 2022. Although, the guideline was created for health care providers, it includes helpful information for you. We have summarized this information for you as "the Bare Bones Podcast Series".

Did you know?

3 Most Common Fracture Sites

Knee Regions and Hip

What is the Bare Bones Podcast Series?

A series of 9 podcasts and related handouts, entitled "The Bare Bones Podcast" will share what you can do to improve your bone health and reduce your fracture risk over time. There are many things you can do for your bone health!

BARE BONES PODCAST — 9 Podcasts & Handouts

1. "Introduction to The Bare Bones of Bone Health"
2. "Fractures 101" — Medical History, Risk Factors, & Falls
3. "Blood Tests: a Window into You" — Blood Screening
4. "I See Your Skeleton" — Bone Mineral Density Measurements
5. "Vitamin D for All, Calcium for Some" — Diet and Supplements
6. "Get Moving and Loading" — Rehabilitation Therapy
7. "Pills or Poisons & Atomic Habits" — Drug Therapy
8. "Snap and Crack" — Fracture Management
9. "Bone Research" — Future Directions

To Do List — Tips For Managing Your Bone Health

- ☐ Listen to the 9 podcast episodes
- ☐ Apply what you learn to your daily life
- ☐ Read the 9 related handouts
- ☐ Discuss what you learned with your health care team

Knowledge Is Power. See The Complete Picture For Healthy Bones & A Fracture-Free Life.

Learn More

SCI Fragments: www.scifragments.ca

The information contained in these handouts & podcast are not intended to replace medical advice. Readers are advised to discuss their individual circumstances with their doctor & rehab care team.

Copyright ©2023,KITE Research Institute-University Health Network, Toronto, Ontario, Canada. This handout may be copied freely, but cannot be altered without permission.

Funding

This project is supported in part by the Paralyzed Veterans of America (PVA) Education Foundation(Grant 867), UHN Foundation, KITE Research Institute & CravenLab.

www.cravenlab.ca

1. Title
2. Episode # and Icon
3. Key Concept
4. "Did You Know?"
5. Background Information
6. Action Item
7. Key Take-Home Message
8. Website and Resource Links
